# Sleep efficiency changes in patients with sleep disorders after inpatient treatment: a real-world retrospective study

**DOI:** 10.3389/fpsyt.2026.1761386

**Published:** 2026-02-06

**Authors:** Jie Ren, Qiuxia Xiao, Jing Zhang, Runxing Ma, Yanyan Ma, Biao Li, Lan Yang, Liulin Xiong

**Affiliations:** 1 Sleep Medicine Center, The Third Affiliated Hospital of Zunyi Medical University (The First People’s Hospital of Zunyi), Zunyi, Guizhou, China; 2 Department of Anesthesiology, The Third Affiliated Hospital of Zunyi Medical University (The First People’s Hospital of Zunyi), Zunyi, Guizhou, China

**Keywords:** sleep disorders, sleep efficiency, inpatient treatment, apnea–hypopnea index, red blood cell count

## Abstract

**Background:**

Sleep disorders are prevalent conditions that affect both physical and mental health. Evidence describing short-term changes in sleep during hospitalization remains limited. In this study, we examined changes in sleep efficiency (SE) among hospitalized patients with sleep disorders in real-world inpatient settings and explored associated factors.

**Methods:**

We retrospectively analyzed electronic medical records of patients with sleep disorders who received inpatient treatment at Sleep Medicine Center of Zunyi First People’s Hospital. Missing data were handled using multiple imputation. The Wilcoxon signed-rank test was used to compare pre- and post-hospitalization SE derived from sleep diaries. LASSO regression was used to identify variables associated with post-treatment SE. Multiple linear regression models and sensitivity analyses were conducted to confirm the robustness of the main results.

**Results:**

SE improved significantly after hospitalization. LASSO regression identified six candidate predictors. The apnea–hypopnea index (AHI) showed a consistent negative relationship with post-treatment SE in the multiple imputation model. Red blood cell count (RBC) was also associated with post-treatment SE in the multiple imputation model, while other variables including triglyceride, monocyte percentage, Pittsburgh Sleep Quality Index, and Insomnia Severity Index showed limited effects.

**Conclusions:**

Short-term inpatient management was associated with significant improvement in diary-derived SE, and baseline AHI showed potential negative correlation with post-treatment SE. Further validation in larger, multi-center cohorts is needed to confirm these findings and better understand the factors influencing sleep improvement in hospitalized patients.

## 1 Introduction

Sleep is fundamental to human physical and psychological health, which plays a crucial role in cognitive function (1), emotion regulation (2), metabolic balance (3) and cardiovascular health (4). With accelerating modern lifestyles and increasing psychological stress, sleep disorders have become increasingly prevalent and are now widely regarded as a major public health concern (5, 6). According to the third edition of the International Classification of Sleep Disorders (ICSD-3), sleep disorders are primarily categorized into six major types, including insomnia disorders, sleep-related breathing disorders, central hypersomnolence disorders, circadian rhythm sleep–wake disorders, sleep-related movement disorders, and parasomnias (7). Sleep disorders not only reduce quality of life but also increase the risk of chronic diseases and healthcare burden (8).

Sleep efficiency (SE), defined as the ratio of total sleep time (TST) to time in bed (TIB), is a widely used indicator of sleep for assessing treatment response and monitoring symptom changes in patients with sleep disorders (9–11). Low SE is regarded as a serious clinical issue and has been associated with poor health conditions (12, 13). Randomized clinical trials have further confirmed that SE is a measurable and responsive outcome in the treatment of insomnia (14, 15). There are still certain limitations in applying these results to actual clinical practice.

In recent years, the real-world perspective has received increasing attention in the sleep disorders research. Emerging studies have explored the application of machine learning models to evaluate the predictive performance of insomnia-related outcomes across different populations (16), highlighting the potential of data-driven approaches in sleep medicine. In parallel, population-based surveys conducted during and after the COVID-19 pandemic have further demonstrated the high prevalence and heterogeneity of sleep disturbances, including insomnia and obstructive sleep apnea, and have identified demographic and clinical factors—such as sex and disease severity—as important correlates of sleep impairment (17). However, evidence derived from real-world inpatient settings remains limited. Previous retrospective studies have primarily focused on cognitive behavioral therapy for insomnia (CBT-I) over one week hospitalization period (18), or have reported exploratory findings regarding the effects of specific interventions on sleep-related indicators (19). Overall, the existing evidence tends to concentrate on specific populations or single intervention scenarios, which restricts their ability to capture dynamic changes in sleep parameters during hospitalization. Importantly, systematic characterization of short-term changes in SE during hospitalization, as well as inter-individual variability in treatment response under routine clinical conditions, remains insufficiently addressed.

Therefore, this study aimed to investigate short-term changes in SE during hospitalization in a real-world inpatient population and to explore potential clinical and laboratory factors associated with these changes. Our findings may provide exploratory, hypothesis-generating evidence to inform future mechanistic investigations and prospective clinical studies.

## 2 Methods

### 2.1 Study population and data collection

This retrospective study included electronic medical record data from patients with sleep disorders who were diagnosed and treated at the Sleep Medicine Center of Zunyi First People’s Hospital (Third Affiliated Hospital of Zunyi Medical University) between March 24, 2025, and August 25, 2025. Exclusion criteria: (1) age ≤ 18 years; (2) comorbid with severe mental disorders or neurological diseases (such as schizophrenia, Parkinson’s disease, etc.); (3) treatment plan interruption or poor compliance. During the hospitalization period, the patients filled out a sleep diary, recording their subjective sleep parameters, including total sleep time (TST), time in bed (TIB), and sleep efficiency (SE). The length of hospitalization for all patients did not exceed 12 days (with an average of 10 days). Treatment compliance was assessed daily by the clinical team based on completion of planned interventions and diary completion. This retrospective study was approved by the Ethics Committee of the First People’s Hospital of Zunyi (Ethical Approval No. (2025)-1-719). All procedures were conducted in accordance with the Declaration of Helsinki and relevant institutional guidelines. Owing to the retrospective design and the use of anonymized clinical data, the requirement for informed consent was waived by the Ethics Committee.

### 2.2 Diagnostic and treatment background

**Figure 1:**
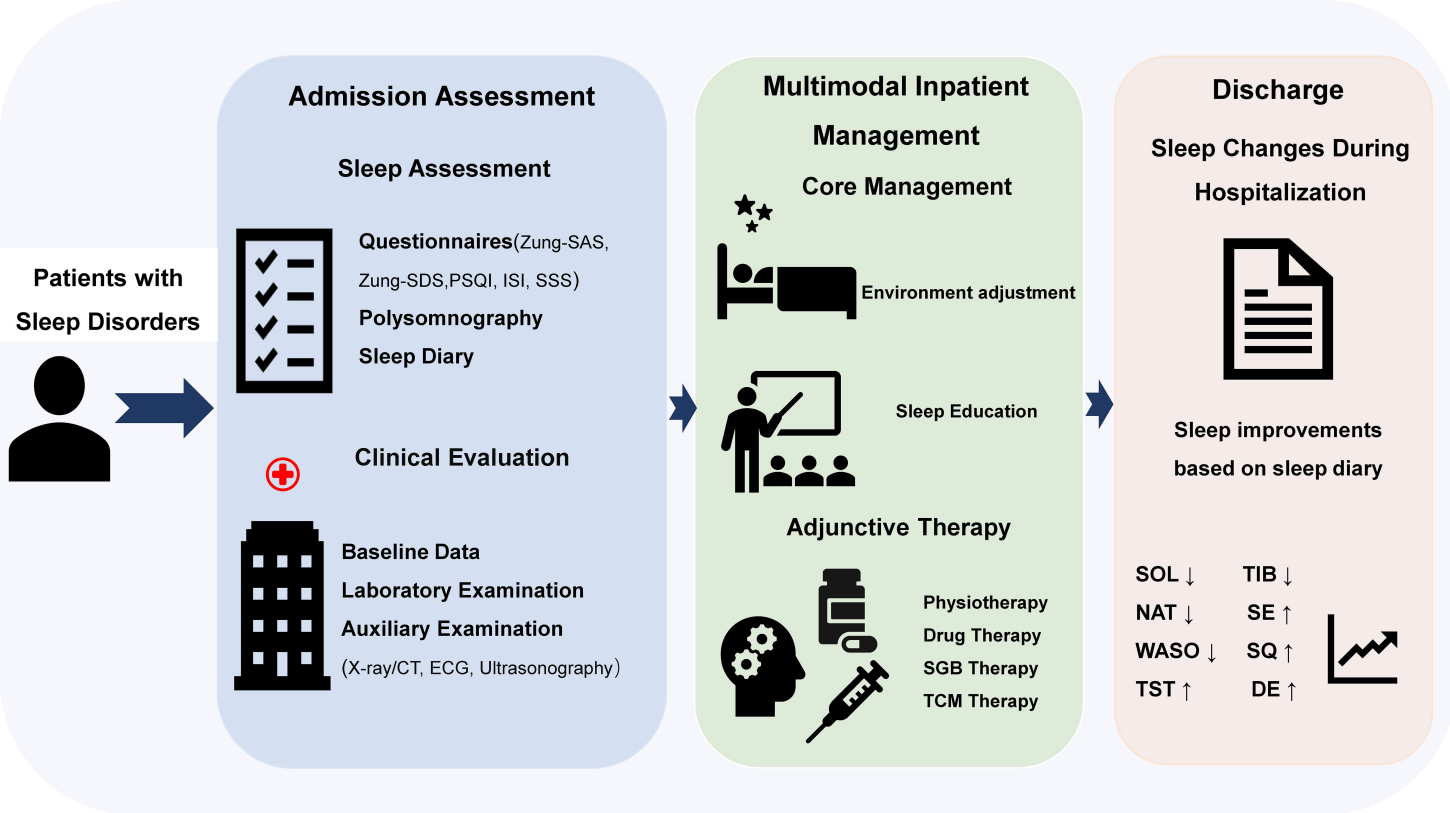
Overview of the comprehensive inpatient intervention program for patients with sleep disorders. DE, Daytime energy; ISI, Insomnia Severity Index; NAT, Night awake times; PSQI, Pittsburgh Sleep Quality Index; SSS, Somatic Self-rating Scale; SE, Sleep efficiency; SGB, Stellate ganglion block; SOL, Sleep onset latency; SQ, Sleep quality; TCM, Traditional Chinese medicine; TIB, Time in bed; TST, Total sleep time; WASO, Wake after sleep onset; Zung-SAS; Zung Self-Rating Anxiety Scale; Zung-SDS, Zung Self-Rating Depression Scale. This program is provided by the same team throughout the entire intervention process.

This exploratory retrospective investigation primarily aimed to estimate overall changes in SE before and after inpatient treatment in a real-world hospitalized population. In routine clinical practice, there is a significant overlap in the symptoms and comorbidities of different sleep disorder subtypes, and the sample sizes are also relatively limited. The analysis was mainly conducted at the overall cohort level. At the same time, we also conducted subgroup analyses to explore the potential heterogeneity associated with the main sleep disorder subtypes. All patients received standardized inpatient management by the same clinical team (**Figure 1**), including environmental adjustment, sleep education, and adjuvant therapy based on clinical assessment. Drug treatment is a part of routine clinical care, but it has not been recorded in a structured manner in detail, making it impossible to make quantitative adjustments.

### 2.3 Data measures

#### 2.3.1 Clinical, laboratory, and sleep-related measures

Clinical and demographic information was extracted from the electronic medical records, including age, gender, body mass index (BMI), retirement status, white blood cells (WBC), red blood cells (RBC), platelets (PLT), hematocrit (HCT), total cholesterol (TC), triglyceride (TG), total protein (TP), neutrophils percentage (NP), lymphocytes percentage (LP), monocytes percentage (MP).

Baseline sleep-related assessments included polysomnography (PSG) parameters and standardized questionnaires. PSG monitoring indicators include sleep onset latency (SOL), rapid eye movement (REM) latency, wakefulness (W period), R period, N1 period, N2 period, N3 period, apnea-hypopnea index (AHI). Questionnaire assessments included the Zung Self-Rating Anxiety Scale (Zung-SAS), Zung Self-Rating Depression Scale (Zung-SDS), Pittsburgh Sleep Quality Index (PSQI), Insomnia Severity Index score (ISI), Somatic Self-rating Scale (SSS). Subjective sleep parameters provided in the sleep diary, SOL, night awake times (NAT), wake after sleep onset (WASO), total sleep time (TST), time in bed (TIB), sleep efficiency (SE), sleep quality (SQ) score, daytime energy (DE) score.

#### 2.3.2 Missing data and multiple imputation

In this study, there were approximately 10% to 20% missing values for some of the variables. To reduce the loss of sample size and potential bias caused by analyzing only complete cases alone, we summarized the proportion and pattern of missing values for each variable (**Supplementary Table S1**). Then we used the multiple imputation by chained equations (MICE) method to handle the missing data. The imputation frequency was set to 50 imputations, with 30 iterations each time to ensure model convergence (20). Imputed datasets were analyzed separately and pooled using Rubin’s rules.

### 2.4 Least absolute shrinkage and selection operator regression analysis

LASSO regression (21) was performed as an exploratory, hypothesis-generating approach for variable selection. Pre-treatment SE was specified *a priori* and retained in subsequent multivariable models as a baseline prognostic factor. Because it is strongly correlated with post-treatment SE, pre-treatment SE was not included in the primary LASSO procedure to avoid redundancy and dominance effects associated with highly correlated predictors (22). LASSO was therefore applied only to the remaining candidate variables. Post-treatment SE was treated as the dependent variable, with pre-treatment SE included as a covariate in multivariable regression models. A sensitivity LASSO analysis including pre-treatment SE was additionally conducted, with results reported in **Supplementary Table S2**.

### 2.5 Sensitivity analyses

To verify the reliability of the results, we conducted two sensitivity analyses. One was a covariance analysis based on the original data, without any imputation processing. The other employed a multiple linear regression method, with the change value of SE (ΔSE= post-treatment SE - pre-treatment SE) serving as the dependent variable. For subgroup analyses, patients were categorized into an insomnia-dominant group and other sleep-related conditions based on the admitting diagnosis documented in the medical record.

### 2.6 Statistical analysis

All analyses were performed using R software (version 4.3.2). Continuous variables were expressed as mean ± standard deviation (SD) or median (interquartile range, IQR), while categorical variables were presented as frequencies (percentages). Normality was tested using the Shapiro-Wilk test. Comparisons of sleep indicators before and after treatment were conducted using paired t-tests or Wilcoxon signed-rank tests.

## 3 Results

### 3.1 Study population and data completeness

**Figure 2:**
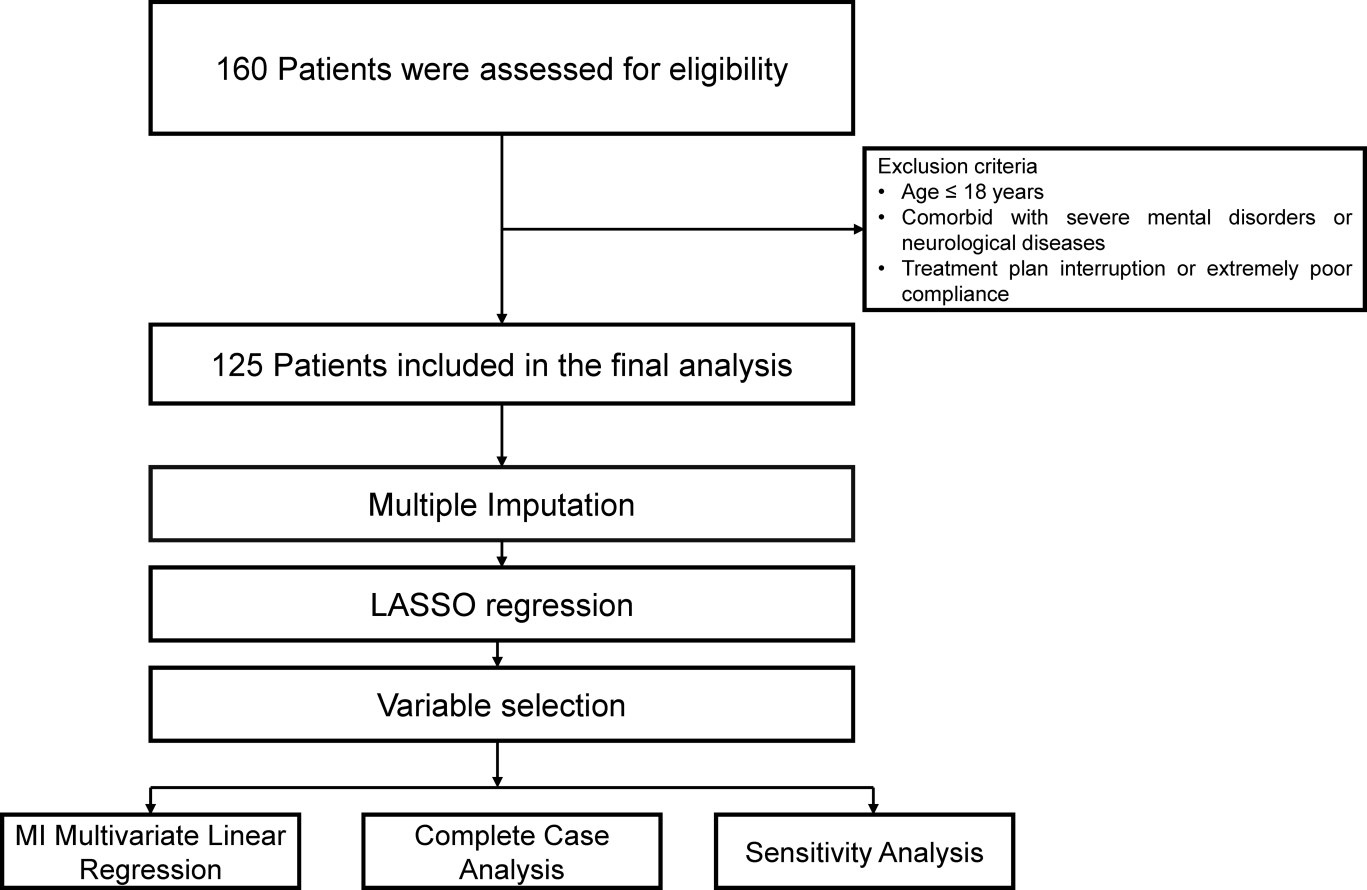
Study population section and analysis procedures. MI, Multiple imputation.

A total of 160 inpatients with sleep disorders were initially screened. After applying the inclusion and exclusion criteria, 125 patients were finally included in the final analysis (**Figure 2**). Baseline characteristics after imputation are summarized (**Table 1**). PSG parameters at baseline reflected heterogeneous sleep architecture and respiratory profiles across the cohort, encompassing SOL, REM latency, wakefulness period (W), REM sleep (R), non-REM sleep stages (N1, N2, and N3), as well as AHI (**Table 1**). The distribution of AHI values indicated varying degrees of respiratory disturbance during sleep, supporting the inclusion of patients with mixed sleep disorder phenotypes. Subjective sleep quality and symptom burden at baseline were assessed using validated questionnaires (**Table 1**). PSQI and ISI scores indicated a generally impaired subjective sleep experience and moderate-to-severe insomnia symptoms in a substantial proportion of patients. In addition, Zung-SAS, Zung-SDS and SSS suggested varying levels of emotional distress and somatic symptom burden at admission. Together, these assessments characterize a clinically heterogeneous inpatient population with combined objective sleep abnormalities and subjective sleep-related complaints at baseline.

**Table 1:** Patient characteristics after multiple interpolations.

Variable	Overall
N	125
Sex (%)	
Female	79 (63.2%)
Male	46 (36.8%)
Age (median [IQR] year)	56 [47.00, 63.00]
Retire (%)	
No	59 (47.2%)
Yes	66 (52.8%)
BMI (median [IQR] kg/m^2^)	22.48 [20.81, 24.22]
WBC (median [IQR]×10^9^/L)	5.40 [4.67, 6.35]
NP (median [IQR] %)	59.00 [51.88, 66.74]
LP (median [IQR] %)	31.66 [25.59, 37.14]
MP (median [IQR] %)	5.70 [4.80, 6.96]
RBC (median [IQR] ×10^12^/L)	4.35 [4.03, 4.73]
HCT (median [IQR]%)	127.5 [116.76, 136.60]
PLT (median [IQR]×10^9^/L)	205 [166.46, 232.24]
TC (median [IQR] mmol/L)	4.79 [4.11, 5.32]
TG (median [IQR] mmol/L)	1.48 [1.05, 2.12]
TP (median [IQR] mmol/L)	67.26 [64.19, 70.19]
Zung-SAS (median [IQR] score)	45.08 [40.00, 53.08]
Zung-SDS (median [IQR] score)	52.96 [44.96, 60.70]
PSQI (median [IQR] score)	16.00 [13.98, 18.00]
ISI (median [IQR] score)	18.80 [15.84, 23.44]
SSS (median [IQR] score)	41.98 [35.00, 48.34]
TST_psg (median [IQR] min)	384.3 [344.63, 414.45]
TIB_psg (median [IQR] min)	512.86 [464.36, 558.42]
SE_psg (median [IQR] %)	77 [69, 85]
WASO_psg (median [IQR] min)	42.94 [23.67, 80.40]
NAT_psg (median [IQR] times)	14.40 [10.24, 21.06]
SOL_psg (median [IQR] min)	13.16 [6.72, 27.32]
REM latency_psg (median [IQR] min)	98.49 [78.83, 147.28]
W_psg (median [IQR] min)	58.47 [38.38, 106.29]
R_psg (median [IQR] min)	75.31 [51.66, 93.50]
N1_psg (median [IQR] min)	37.39 [24.57, 55.35]
N2_psg (median [IQR] min)	245.08 [203.49, 275.63]
N3_psg (median [IQR] min)	8.83 [1.80, 31.87]
AHI_psg (median [IQR] events/h)	8.05 [2.96, 18.94]
CBT-I (%)	
No	41 (32.8%)
Yes	84 (67.2%)

AHI, Apnea-Hypopnea Index; BMI, Body Mass Index; CBT-I, Cognitive Behavioral Therapy for Insomnia; DE, Daytime Energy; HCT, Hematocrit; ISI, Insomnia Severity Index; LP, Lymphocytes percentage; MP, Monocyte percentage; NAT, Night awake times; NP, Neutrophilic percentage; PLT, Platelet; psg, polysomnography; PSQI, Pittsburgh Sleep Quality Index; RBC, Red blood cell; REM, Rapid eye movement; SOL, Sleep onset latency; SQ, Sleep quality; SSS, Somatic Self-rating Scale; TC, Total cholesterol; TG, Triglyceride; TIB, Time in bed; TP, Total protein; TST, Total sleep time; SE, Sleep efficiency; WASO, Wake after sleep onset; WBC, White blood cell; Zung-SAS, Zung Self-Rating Anxiety Scale; Zung-SDS, Zung Self-Rating Depression Scale.

### 3.2 Changes in sleep diary parameters

**Figure 3:**
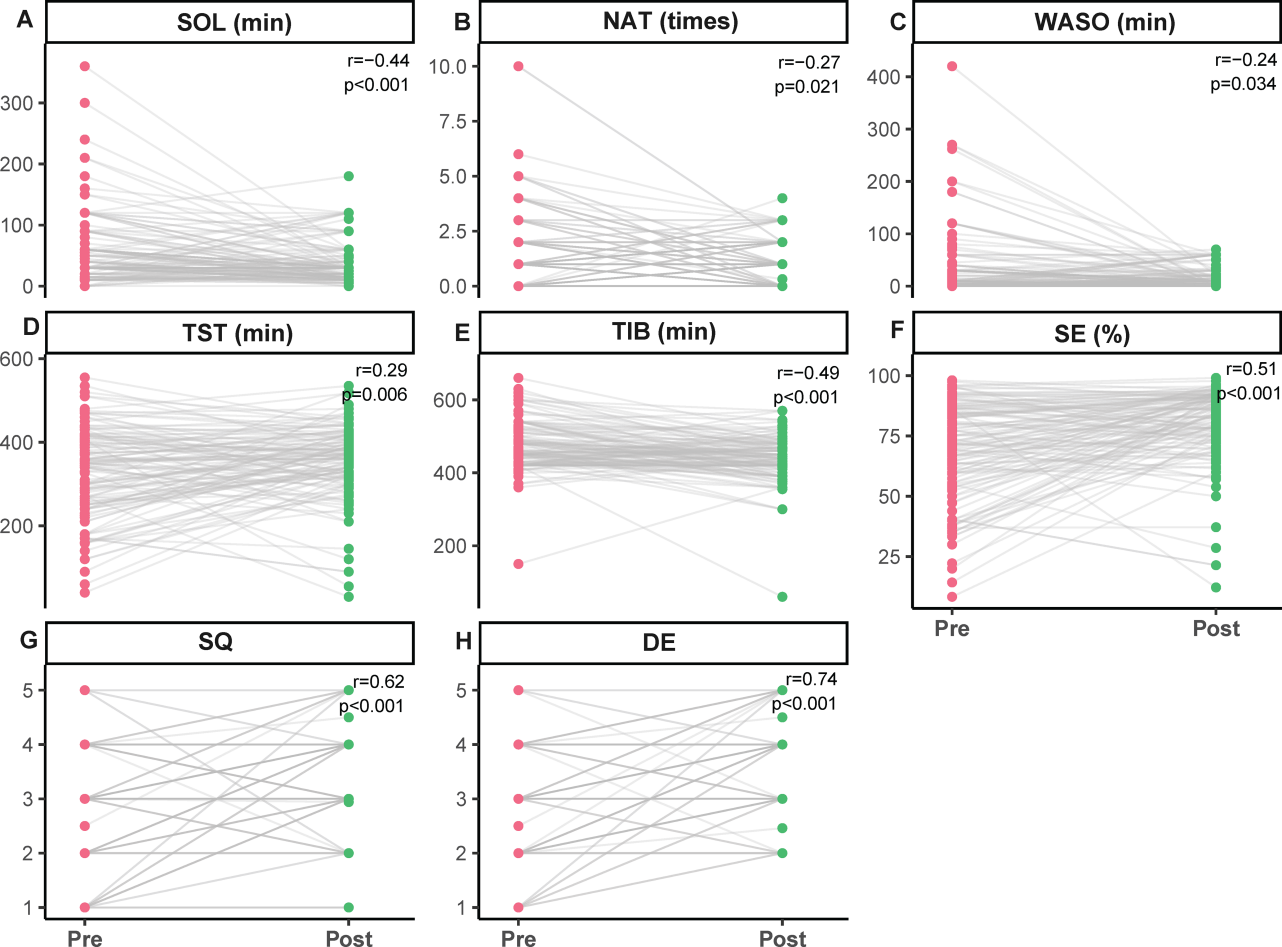
Comparison of sleep diary parameters before and after treatment. **(A)** Sleep onset latency (SOL). **(B)** Night awake times (NAT). **(C)** Wake after sleep onset (WASO). **(D)** Total sleep time (TST). **(E)** Time in bed (TIB). **(F)** Sleep efficiency (SE). **(G)** Sleep quality (SQ). **(H)** Daytime energy (DE). Each point represents one patient. The grey line connects the pre-treatment (red) and post-treatment (green) measurements. The Hodges–Lehmann estimator (HL) was used to estimate the median difference, and the corresponding p-value from the Wilcoxon signed-rank test is presented. The estimated median difference (HL) and its 95% confidence interval are rounded to two decimal places.

Sleep diary data from 125 patients were analyzed using Wilcoxon signed-rank test after multiple imputation. Several sleep indicators showed varying degrees of changes after hospitalization treatment (**Table 2**, **Figure 3**). SOL was significantly shortened (HL = -20.00, 95% CI, -27.50 to -10.00, *p* < 0.001, *r* = -0.44) (**Table 2**, **Figure 3A**). NAT showed a modest decrease (HL = -0.50, 95% CI, -1.00 to 0.00, *p* = 0.021, *r* = -0.27) (**Table 2**, **Figure 3B**). WASO also decreased (HL = -7.50, 95% CI, -17.50 to 0.00, *p* = 0.034, *r* = -0.24) (**Table 2**, **Figure 3C**). TST increased significantly (HL = 29.00, 95% CI, 9.00 to 48.50, *p* = 0.006, *r* = 0.29) (**Table 2**, **Figure 3D**), while TIB was significantly reduced (HL = -35.00, 95% CI, -48.50 to -20.00, *p* < 0.001, *r* = -0.49) (**Table 2**, **Figure 3E**). SE also increased (HL = 10.00, 95% CI, 6.00 to 14.00, *p* < 0.001, *r* = 0.51) (**Table 2**, **Figure 3F**). SQ and DE scores showed more pronounced improvements (HL = 1.00, 95% CI, 0.75 to 1.50, *p* < 0.001; HL = 1.00, 95%, 1.00 to 1.50, *p* < 0.001), with relatively larger effect sizes (*r* = 0.62, *r* = 0.74), suggesting that perceived improvements were more prominent than changes in objective sleep measures (**Table 2**, **Figure 3G, H**).

**Table 2:** Pre–Post changes in sleep diary parameters among patients with sleep disorders.

Variables	Pre_treatment	Post_treatment	HL_CI	p	r
SOL (min)	30.00 [20.00, 60.00]	30.00 [15.00, 40.00]	-20.00 [-27.50, -10.00]	<0.001	-0.44
NAT (times)	1.00 [0.00, 2.00]	1.00 [0.00, 2.00]	-0.50 [-1.00, -0.00]	0.021	-0.27
WASO (min)	10.00 [0.00, 30.00]	10.00 [0.00, 20.00]	-7.50 [-17.50, -0.00]	0.034	-0.24
TST (min)	345.00 [250.00, 405.00]	370.00 [320.00, 408.00]	29.00 [9.00, 48.50]	0.006	0.29
TIB (min)	455.00 [425.00, 510.00]	440.00 [418.00, 480.00]	-35.00 [-48.50, -20.00]	<0.001	-0.49
SE (%)	72 [57, 86]	85 [74, 91]	10 [6, 14]	<0.001	0.51
SQ (score)	3.00 [2.00, 4.00]	4.00 [3.00, 4.00]	1.00 [0.75, 1.50]	<0.001	0.62
DE (score)	3.00 [2.00, 4.00]	4.00 [3.00, 4.00]	1.00 [1.00, 1.50]	<0.001	0.74

DE, Daytime energy; HL, Hodges–Lehmann median differences; NAT, Night awake times; SE, Sleep efficiency; SOL, Sleep onset latency; SQ, Sleep quality; TIB, Time in bed; TST, Total sleep time; WASO, Wake after sleep onset.

### 3.3 Variable selection using LASSO regression

**Figure 4:**
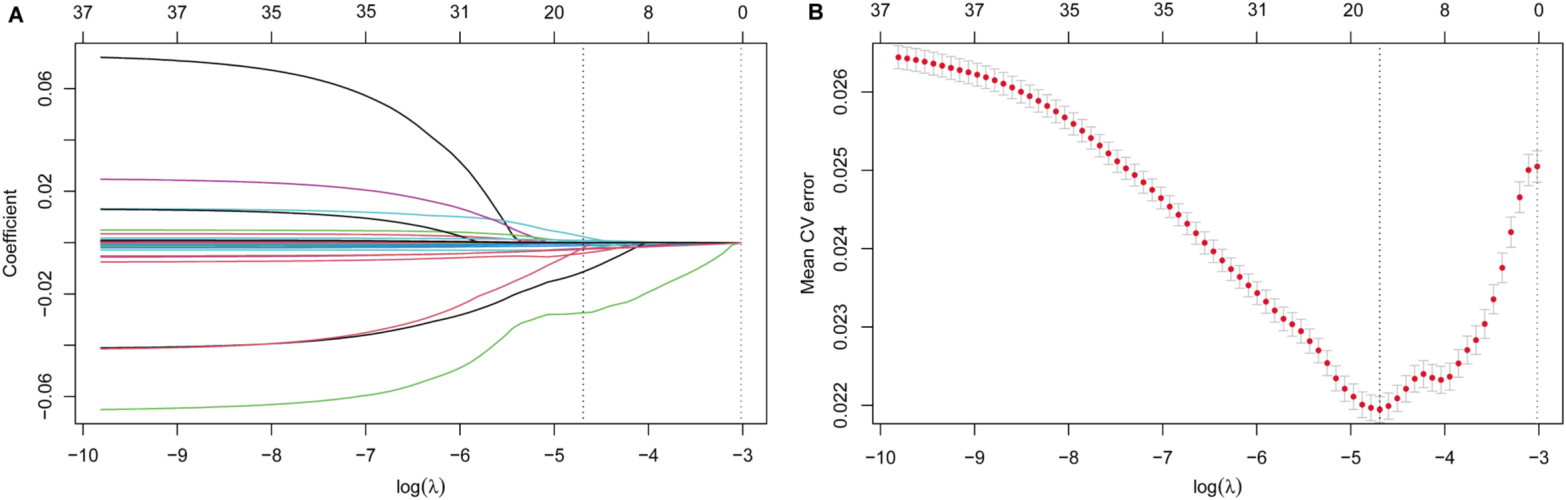
LASSO regression analysis of potential prediction variables. **(A)** The coefficient profile plot was produced against the Log Lambda sequence. **(B)** Cross validation plot for the penalty term.

LASSO regression model was applied to identify candidate variables associated with post-treatment SE, aiming to mitigate multicollinearity and enhance the model stability in the context of a limited sample size. Through 10-fold cross-validation, the optimal penalty coefficient (λ) was determined to be 0.009167 (**Figures 4A, B**). Under this penalization, 16 non-zero coefficient variables were retained (**Supplementary Table S2**).

### 3.4 Multivariable regression analysis

**Figure 5:**
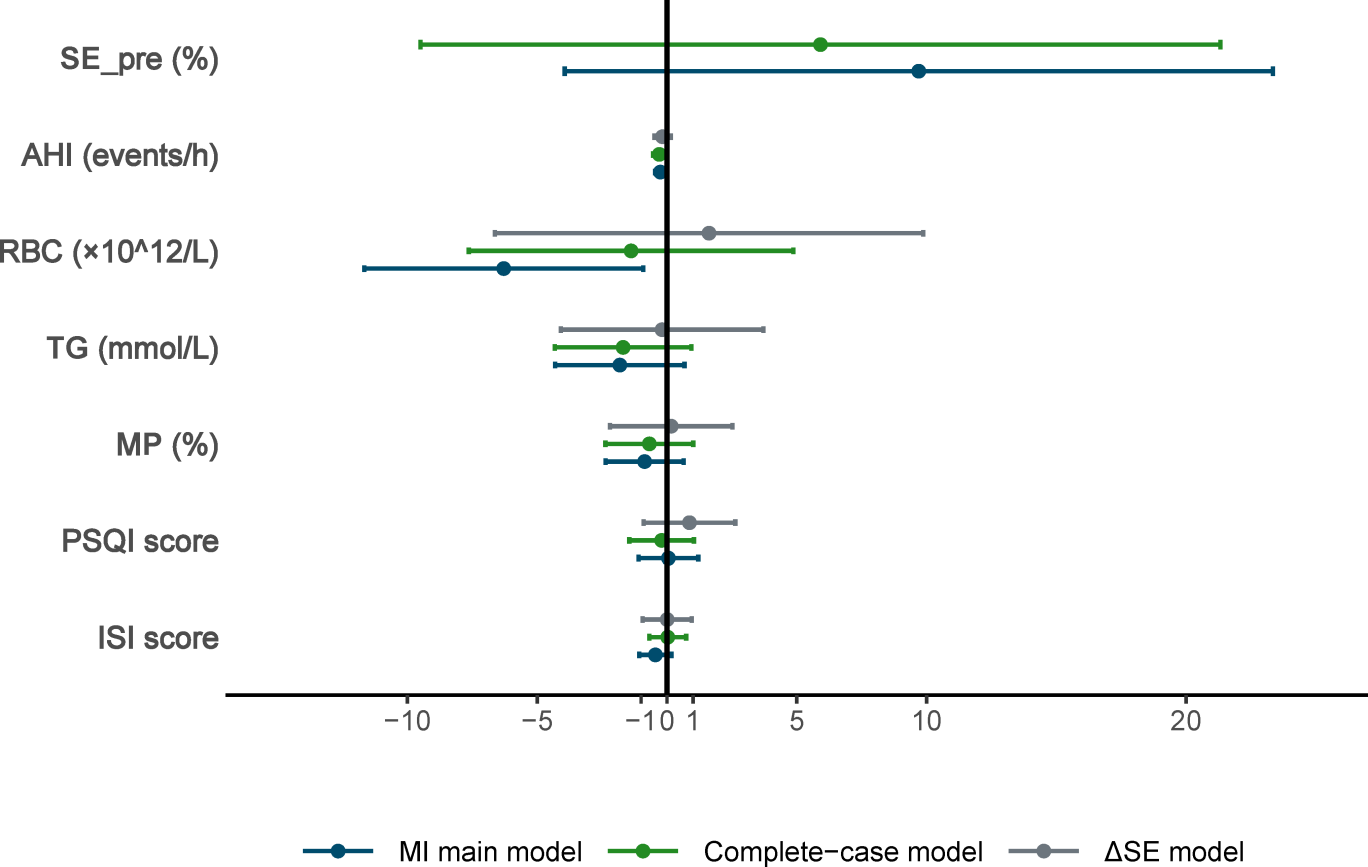
Forest plot of multivariate linear regression and sensitivity analysis. AHI, Apnea Hypopnea Index; CC, Complete case; ISI, Insomnia Severity Index; MI, Multiple interpolation; MP, Monocytes percentage; PSQI, Pittsburgh Sleep Quality Index; RBC, Red blood cell; TG, Triglyceride; ΔSE, Post-treatment SE-Pre-treatment SE.

Based on the magnitude of the coefficients, clinical relevance, and data completeness, baseline AHI, RBC, Triglyceride (TG), monocyte percentage (MP), AHI, PSQI score, and ISI score were included in multivariable linear regression model after multiple imputation (**Table 3**, **Figure 5**). Results from a sensitivity LASSO analysis including pre-treatment SE are presented **Supplementary Table S2**. The main variable selection results were largely consistent with the primary analysis, particularly with respect to the association between AHI and post-treatment SE.

**Table 3:** Multivariate linear regression and sensitivity analysis.

Variable	β_MI	95% CI	95% CI	p_MI	β_CC	95% CI	95% CI	p_CC	β_ΔSE	95% CI	95% CI	p_ΔSE
		Lower	Upper			Lower	Upper			Lower	Upper	
SE_pre (%)	9.70	-3.95	23.34	0.162	5.91	-9.50	21.32	0.447				
AHI(events/h)	-0.26	-0.45	-0.06	0.011	-0.30	-0.53	-0.07	0.013	-0.17	-0.49	0.14	0.274
RBC (×10^12^)	-6.29	-11.66	-0.92	0.022	-1.38	-7.64	4.87	0.661	1.62	-6.64	9.87	0.698
TG (mmol/L)	-1.82	-4.31	0.68	0.151	-1.69	-4.32	0.94	0.205	-0.19	-4.10	3.71	0.923
MP (%)	-0.86	-2.37	0.64	0.258	-0.68	-2.37	1.01	0.426	0.16	-2.20	2.52	0.891
PSQI score	0.06	-1.09	1.21	0.925	-0.21	-1.45	1.04	0.742	0.86	-0.90	2.63	0.335
ISI score	-0.45	-1.07	0.17	0.156	0.03	-0.68	0.74	0.935	0.01	-0.94	0.95	0.991

AHI, Apnea-hypopnea index; CC, Complete Case; ISI, Severity Index score; MI, Multiple interpolation; MP, monocytes percentage; PSQI, Pittsburgh Sleep Quality Index; RBC, red blood cell; TG, triglyceride; ΔSE, Post-treatment SE-Pre-treatment SE.

In the primary imputed model, higher baseline AHI was significantly correlated with the improvement in SE during hospitalization (β = -0.26, 95% CI, -0.45 to -0.06, *p* = 0.011). RBC was also negatively associated with SE change (β = -6.29, 95% CI, -11.66 to -0.92, *p* = 0.022). TG (β = -1.82, 95% CI, -4.31 to 0.68, *p* = 0.151) and MP (β = -0.86, 95%CI, -2.37 to 0.64, *p* = 0.258) showed the same negative association but didn’t reach statistical significance. Baseline PSQI (β = 0.06, 95% CI, -1.09 to 1.21, *p* = 0.925) and ISI (β = -0.45, 95% CI, -1.07 to 0.17, *p* = 0.156) were not significantly correlated with SE changes. Pre-treatment SE demonstrated a mild but non-significant positive association (β = 9.7, 95%CI, -3.95 to 23.34, p = 0.162), consistent with its role as a covariate (**Table 3**, **Figure 5**).

### 3.5 Sensitivity and subgroup analyses

In the complete-case model, baseline AHI remained significantly associated with SE changes (β = -0.30, 95% CI, -0.53 to -0.07, *p* = 0.013). RBC results showed no statistically significant association (β = -1.38, 95% CI, -7.64 to 4.87, *p* = 0.661). TG (β = -1.69, 95%CI, -4.32 to 0.94, *p* = 0.205) and MP (β = -0.68, 95%CI, -2.37 to 1.01, *p* = 0.426) remained in the same negative direction but also did not reach statistical significance (**Table 3**, **Figure 5**). In the ΔSE model (ΔSE= Post-treatment SE - Pre-treatment SE), the direction of the association between AHI and SE change remained consistent without statistical significance (β = -0.17, 95% CI, -0.49 to 0.14, *p* = 0.274) (**Table 3**, **Figure 5**). No other variables showed statistically significant associations with ΔSE. Subgroup analyses stratified by dominant sleep disorder category (insomnia-dominant *n* = 104 versus other sleep-related conditions, *n* = 21) yielded directionally consistent results for AHI across subgroups. An association between pre-treatment SE and post-treatment SE was observed only in the insomnia-dominant subgroup (**Supplementary Table S3**).

## 4 Discussion

In this retrospective study, we investigated the changes in sleep parameters of inpatients with sleep disorders during their short-term hospitalization. The results showed that several sleep indicators based on sleep diaries improved after hospitalization, including SOL, WASO, TST, and SE. This suggests that comprehensive inpatient management during short-term hospitalization may be associated with an improvement in overall sleep conditions. Further analysis revealed that baseline AHI and some routine laboratory indicators, such as RBC, TG, and MP, were associated with the changes in SE. These associations suggest that inter-individual variability in respiratory and physiological status at admission may be related to differential short-term sleep responses during hospitalization.

Several factors inherent to the inpatient setting may contribute to the observed improvement in SE. The regular schedule and less disturbed sleep environment during hospitalization may provide favorable conditions for the improvement of SE. In addition, temporary relief from family- and work-related stressors during hospitalization may reduce hyperarousal, a key mechanism underlying the maintenance of sleep disorders (23). Patients also received non-pharmacological interventions, including sleep hygiene education, CBT-I and relaxation training, all of which have been shown in previous studies to improve sleep quality (24–26). Moreover, according to clinical needs, some patients received auxiliary interventions such as physical therapy (27), TCM treatment (28) or SGB (29), which may further influence sleep through pathways related to autonomic regulation or inflammatory processes.

Importantly, although statistically significant improvements in SE were observed in our findings, the clinical relevance of the magnitude of change warrants cautious interpretation. At present, there is no universally accepted minimal clinically important difference (MCID) for SE, particularly in heterogeneous real-world inpatient populations (9, 30, 31). In clinical practice and sleep research, SE is commonly used as an indicator of sleep continuity (9) and values around 85% are often used as a practical reference for relatively good sleep quality rather than a strict clinical threshold (30). Previous studies emphasize that changes in SE should be interpreted in conjunction with insomnia symptoms and daytime functional outcomes, rather than judged solely against predefined quantitative cutoffs (30, 31). Given the absence of a control group and standardized clinical thresholds in the present study, our findings should be regarded as exploratory and hypothesis-generating rather than confirmatory evidence of clinically meaningful improvement.

It should be noted that our primary focus was on the changes in sleep parameters under comprehensive inpatient treatment, and we were unable to distinguish the independent contributions of the intervention measures. We would like to acknowledge that SE in this study was primarily derived from daily sleep diaries, which are subject to recall and expectation biases. However, sleep diaries are widely used and recommended in insomnia research and clinical practice as reliable tools for evaluating short-term changes in sleep continuity and treatment response (32, 33). PSG is the gold standard for objective sleep assessment (34), repeated PSG measurements during short-term hospitalization are often impractical in routine clinical practice. Therefore, the use of sleep diaries represents a pragmatic approach aligned with real-world clinical practice. Among PSG-derived parameters, AHI was selected for further modeling based on its clinical relevance and data completeness, while other sleep architecture variables were primarily used to characterize baseline sleep status. AHI is a core indicator of nocturnal respiratory stability and sleep-disordered breathing severity (35). Across all analytical models, baseline AHI showed a consistent negative association with SE changes, suggesting that greater respiratory instability at admission may act as an independent correlate of poorer short-term SE improvement during hospitalization. A large-scale population study (35) has demonstrated that the physiological impact of AHI does not emerge beyond a single pathological threshold; rather, its adverse effects increase along a continuous gradient, with progressively greater disruption as AHI increases. Previous studies have shown that even mild respiratory instability may contribute to sleep fragmentation and intermittent hypoxia (36), and clinical studies have further reported that mild to moderate elevations in AHI can weaken the therapeutic efficacy of CBT-I (37). These observations together with our findings support that higher baseline AHI might constrain the extent of SE improvement under comprehensive inpatient management.

Several routine laboratory indicators, such as RBC, TG and MP, were also negatively correlated with the degree of SE improvement, although these relationships were weak and did not consistently reach statistical significance. Notably, all laboratory values were within normal clinical ranges. These markers are not sleep-specific biomarkers, and their associations with SE should therefore be interpreted cautiously. Prior studies suggest inter-individual variability in hematological and metabolic indices within normal range may reflect differences in baseline physiological or inflammatory status rather than overt disease (38, 39). From a clinical perspective, such baseline physiological differences may partly influence an individual’s short-term responsiveness to comprehensive inpatient management, rather than representing direct mechanistic pathways affecting SE.

There was a weak and unstable association between the SE improvement after hospitalization and baseline subjective sleep scales, including PSQI and ISI. This may be related to the intrinsic characteristics of these subjective scales. Both PSQI and ISI reflect not only sleep-related symptoms but are also strongly influenced by emotional burden, anxiety and subjective distress (40). In addition, the inclusion of heterogeneous sleep disorder subtypes with differing treatment responsiveness may further attenuate the prognostic value of baseline subjective measures (41). Therefore, relying solely on the baseline subjective scales may be insufficient to determine the patient’s response to comprehensive intervention in clinical practice, underscoring the need for a more comprehensive assessment combining objective data and clinical characteristics.

This study has several limitations to be acknowledged. First, the retrospective, single-center design without a control group limits causal inference and may affect the generalizability of the findings. Improvements in SE observed during hospitalization may reflect both treatment effects of inpatient management and nonspecific factors such as regression to the mean. Second, SE was primarily assessed using sleep diaries, which, although widely used in clinical research, are subject to recall bias and expectation effects. Third, baseline laboratory parameters were measured only once and therefore do not capture dynamic physiological changes during hospitalization. In addition, several potentially relevant clinical factors, including disease duration, prior treatment history, medication use, and comorbidities, could not be comprehensively incorporated due to limitations of retrospective data extraction. Finally, the clinical heterogeneity among different subtypes of sleep disorders may have influenced the research results. Therefore, future larger-scale, multi-center, prospective studies with longitudinal measurements are warranted to verify these findings.

In conclusion, this real-word study demonstrates that comprehensive inpatient management may lead to short-term improvements in SE and subjective sleep experience. Baseline AHI exhibited a stable negative correlation with SE improvement across different analytical models, highlighting respiratory stability as a potentially important factor limiting short-term treatment response during hospitalization.

## Data Availability

All original data generated or analyzed in this study are provided in the results and **Supplementary Materials**. Requests to access the datasets should be directed to xiongliulin@zmu.edu.cn.
