# Supplementary material for: Sleep efficiency changes in patients with sleep disorders after inpatient treatment: a real-world retrospective study
Source: Front Psychiatry. 2026 Feb 6;17:1761386. doi: 10.3389/fpsyt.2026.1761386 (PMC12933648; doi:10.3389/fpsyt.2026.1761386)
Supplement: Supplementary file 1 [file DataSheet1.docx]

***Supplementary Material***

**Table S1. Summary of missingness and imputation methods for key clinical and polysomnographic variables**

| **Variable** | **Missing(%)** | **Data Type** | **Imputation** | **Notes** |
| --- | --- | --- | --- | --- |
|  |  |  | **Method** |  |
| TST_psg | 19.2 | Continuous | PMM | PSG-derived |
| TIB_psg | 19.2 | Continuous | PMM | PSG-derived |
| SE_psg | 19.2 | Continuous | PMM | PSG-derived |
| WASO_psg | 19.2 | Continuous | PMM | PSG-derived |
| NAT_psg | 19.2 | Continuous | PMM | PSG-derived |
| SOL_psg | 19.2 | Continuous | PMM | PSG-derived |
| REMlatency_psg | 19.2 | Continuous | PMM | PSG-derived |
| W_psg | 19.2 | Continuous | PMM | PSG-derived |
| R_psg | 19.2 | Continuous | PMM | PSG-derived |
| N1_psg | 19.2 | Continuous | PMM | PSG-derived |
| N2_psg | 19.2 | Continuous | PMM | PSG-derived |
| N3_psg | 19.2 | Continuous | PMM | PSG-derived |
| AHI | 19.2 | Continuous | PMM | PSG-derived |
| SSS | 8.8 | Continuous | PMM | Psychological scale |
| TC | 8 | Continuous | PMM | Laboratory |
| TG | 8 | Continuous | PMM | Laboratory |
| ISI | 6.4 | Continuous | PMM | Psychological scale |
| TP | 3.2 | Continuous | PMM | Laboratory |
| Zung-SAS | 3.2 | Continuous | PMM | Psychological scale |
| Zung-SDS | 3.2 | Continuous | PMM | Psychological scale |
| PSQI | 3.2 | Continuous | PMM | Psychological scale |
| WASO_post | 2.4 | Continuous | PMM | Sleep diary |
| BMI | 1.6 | Continuous | PMM | Electronic medical record |
| WBC | 0.8 | Continuous | PMM | Laboratory |
| NP | 0.8 | Continuous | PMM | Laboratory |
| LP | 0.8 | Continuous | PMM | Laboratory |
| MP | 0.8 | Continuous | PMM | Laboratory |
| RBC | 0.8 | Continuous | PMM | Laboratory |
| HCT | 0.8 | Continuous | PMM | Laboratory |
| PLT | 0.8 | Continuous | PMM | Laboratory |
| WASO_pre | 0.8 | Continuous | PMM | Sleep diary |
| TST_pre | 0.8 | Continuous | PMM | Sleep diary |
| SE_pre | 0.8 | Continuous | PMM | Sleep diary |
| NAT_post | 0.8 | Continuous | PMM | Sleep diary |
| DSQ_post | 0.8 | Continuous | PMM | Sleep diary |
| DE_post | 0.8 | Continuous | PMM | Sleep diary |
| Sex | 0 | Binary | Logistic | Electronic medical record |
| Age | 0 | Continuous | PMM | Electronic medical record |
| Retire | 0 | Binary | Logistic | Electronic medical record |
| SOL_pre | 0 | Continuous | PMM | Sleep diary |
| NAT_pre | 0 | Continuous | PMM | Sleep diary |
| TIB_pre | 0 | Continuous | PMM | Sleep diary |
| DSQ_pre | 0 | Continuous | PMM | Sleep diary |
| DE_pre | 0 | Continuous | PMM | Sleep diary |
| SOL_post | 0 | Continuous | PMM | Sleep diary |
| TST_post | 0 | Continuous | PMM | Sleep diary |
| TIB_post | 0 | Continuous | PMM | Sleep diary |
| SE_post | 0 | Continuous | PMM | Sleep diary |
| CBT-I | 0 | Binary | Logistic | Electronic medical record |
| Diagnosis | 0 | Categorical | Logistic | Electronic medical record |

**Abbreviations:** AHI, Apnea-Hypopnea Index, BMI, Body mass index, CBT-I, Cognitive Behavioral Therapy for Insomnia, DE, Daytime energy, HCT, Hematocrit, ISI, Insomnia Severity Index, LP, Lymphocytes percentage, MP, Monocyte percentage, NAT, Night awake times, NP, Neutrophilic percentage, PLT, Platelet, PMM, Predictive mean matching, pre, pre-treatment, psg, polysomnography, PSQI, Pittsburgh Sleep Quality Index, RBC, Red blood cell, REM, Rapid eye movement, SOL, Sleep onset latency, SQ, Sleep quality, SSS, Somatic Self-rating Scale, TC, Total cholesterol, TIB, Time in bed, TG, Triglyceride, TP, Total protein, TST, Total sleep time, SE, Sleep efficiency, WASO, Wake after sleep Onset, WBC, White blood cell, Zung-SAS, Zung Self-Rating Anxiety Scale, Zung-SDS, Zung Self-Rating Depression Scale

**Table S2. LASSO regression for data screening**

| **Variable** | **Coefficient** |  | **Variable** | **Coefficient** |
| --- | --- | --- | --- | --- |
| **Excluding SE_pre** |  |  | **Including SE_pre** |  |
| RBC | -0.02737 |  | RBC | -0.02949 |
| TG | -0.01137 |  | SE_pre | 0.01878 |
| MP | -0.00415 |  | TG | -0.00859 |
| AHI | -0.00258 |  | MP | -0.00286 |
| PSQI | -0.00247 |  | ISI | -0.00241 |
| ISI | -0.00233 |  | AHI | -0.00194 |
| WBC | 0.00229 |  | HCT | -0.00097 |
| DE_pre | -0.00199 |  | N1_psg | 0.00063 |
| HCT | -0.00113 |  | R_psg | -0.00046 |
| N1_psg | 0.00084 |  | WASO_psg | -0.00028 |
| R_psg | -0.00043 |  | Zung-SDS | -0.00023 |
| W_psg | -0.00027 |  | WBC | 0.00019 |
| LP | -0.00025 |  | TST_pre | 0.00012 |
| TST_pre | 0.00015 |  | W_psg | -0.00005 |
| WASO_psg | -0.00015 |  | TST_psg | 0.00004 |
| Zung-SDS | -0.00014 |  | LP | -0.00003 |

**Abbreviations:** AHI, Apnea Hypopnea Index, DE, Daytime energy, HCT, Hematocrit, ISI, Insomnia Severity Index, LP, Lymphocytes percentage, MP, Monocyte percentage, pre, pre-treatment, psg , polysomnography, PSQI, Pittsburgh Sleep Quality Index, RBC, Red blood cell, SE, sleep efficiency, SSS, Somatic Self-rating Scale, TG, Triglyceride, TST, Total sleep time, WASO, Wake after sleep onset, WBC, White blood cell, Zung-SDS, Zung Self-Rating Depression Scale

**Table S3.** **Subtype-specific associations of AHI and pre-treatment SE with post-treatment SE based on multiple imputation analysis**

| **Subtype** | **Variable** | **β_MI** | **95%CI** | **95%CI** | **p_MI** |
| --- | --- | --- | --- | --- | --- |
|  |  |  | **Lower** | **Upper** |  |
| **Insomnia-dominant** | AHI | -0.21 | -0.4 | -0.01 | 0.039 |
| **sleep disorders (n=104)** |  |  |  |  |  |
| **Insomnia-dominant** | SE_pre | 23.6 | 9.97 | 37.23 | 0.001 |
| **sleep disorders (n=104)** |  |  |  |  |  |
| **Other sleep-related** | AHI | -1.08 | -1.92 | -0.25 | 0.015 |
| **conditions (n=21)** |  |  |  |  |  |
| **Other sleep-related** | SE_pre | 8.06 | -4.55 | 29.39 | 0.655 |
| **conditions (n=21)** |  |  |  |  |  |

**Abbreviations:** AHI, apnea-hypopnea index, pre, pre-treatment, SE, SE, Sleep efficiency
